# Awareness of Food Allergies and Allergens on the Menus of Restaurants and Cafes among Saudi Female University Students

**DOI:** 10.3390/healthcare11091259

**Published:** 2023-04-28

**Authors:** Ghzail M. Aljameel, Sahar Abdulaziz AlSedairy, Manal Abdulaziz Binobead, Maha H. Alhussain, Manar Abdulaziz Bin Obaid, Laila Naif Al-Harbi, Ghedeir M. Alshammari, Shaista Arzoo

**Affiliations:** Department of Food Science and Nutrition, College of Food and Agricultural Sciences, King Saud University, Riyadh 11451, Saudi Arabia

**Keywords:** food allergies, awareness, restaurant, cafe, menu

## Abstract

Exposure to allergens could be life-threatening for people with food allergies. Restaurants and cafes are challenging environments for accommodating food allergies. This study aimed to measure King Saud University female students’ awareness about food allergens on restaurants’ and cafes’ menus. This cross-sectional study was conducted on 379 students aged 18 years and above. A paper-based questionnaire was used, which comprised 16 questions related to the definition of food allergies, food allergens, and food allergy symptoms; the definition and prevention of cross-contact of food allergies; emergency treatment procedures for food allergies; strategies for the prevention of food allergy reactions; customer expectations towards restaurants; and preventive measures taken for food allergies. The results indicate that the overall average score of food allergen awareness was 10.90, which falls in the higher range. Furthermore, female students in the age groups of 23 to 27 years and 33 to 37 years had higher levels of awareness than female students in the age group of 18 to 22 years. The results also showed that the level of awareness among science college and health college students was statistically significantly higher (*p* < 0.05) than that among humanities college students. Post-graduate students also showed a higher level of awareness of food allergens than bachelor’s students. These findings also indicate that listing all allergens in the restaurants and cafes’ menus statistically significantly (*p* < 0.05) increased the level of awareness of female students about food allergens on restaurants’ and cafes’ menus, compared to restaurants and cafes that do not list all allergens on their menus. In general, female students at King Saud University showed a high level of awareness about food allergies on restaurants’ and cafes’ menus. The study recommends assessing the impact of awareness of female students with and without food allergies on their practices and behaviors.

## 1. Introduction

Food allergies (FAs) are defined as an adverse reaction of the immune system when consuming food or food ingredients [1,2]. Although almost all foods can provoke an allergic reaction, milk, eggs, fish, shellfish, wheat, nuts, peanuts, and soybeans cause nearly 90% of reported reactions [3]. The skin prick test (SPT), intradermal test, and patch test are the various skin test methods (in vivo) used for the detection of IgE antibodies, but due to the simplicity, rapidity, and low cost, SPT is the most frequently used method [4]. Singleplex, multiparameter, or multiplex immunoassays are the various modern immunoassay methods used for the in vitro detection of serum-specific IgE to allergen molecules [5]. In the singleplex specific IgE immunoassays, only one analyte is measured per analysis. They determine serum-specific IgE using either liquid-phase allergens (i.e., chemiluminescence immunoassay) or solid-phase coupled allergens (i.e., fluorescence enzyme immunoassay) [5,6]. Light-initiated chemiluminescence assay (LICA) has been successfully used for the quantitation of food allergen–specific immunoglobulin E (sIgE) but not for inhaled allergen-sIgE. Recently a method was successfully established for the quantitation of Artemisia-sIgE based on LICA, and this assay was successfully applied in 64 human serum samples, showing good specificity and sensitivity (100% and 82.20%, respectively) [7]. Multiparameter tests (such as the line blot immunoassay) detect specific IgE against a few allergen components at once, usually about 10 (2–11 recombinant or native molecules), along with specific IgE against several natural aeroallergen extracts, while multiplex specific IgE immunoassays (such as the microarray-based immunoassay on immuno solid-phase allergen chip) allow the characterization of IgE sensitization against a broad array of preselected allergens (more than 100 allergens from various allergen sources) [5,6]. An array-based method is an emerging in vitro diagnostic tool for efficiently recognizing patients with adverse reactions to multiple food components. The patient-friendly allergen nanobead array and the macroarray nanotechnology-based immunoassay are the two new multiplex nanotechnology-based immunoassays widely used for molecular allergy exploration [6].

FAs have various symptoms that may appear on the skin, such as itching, urticaria, eczema, vascular oedema, and dermatitis. In addition to impacts on the digestive system, symptoms such as vomiting, abdominal cramps, nausea, diarrhea, and respiratory disorders such as rhinitis, asthma, larynx oedema, and anaphylaxis [8,9] are the most severe consequences caused by particular foods. They may appear within minutes or hours. If the patient does not receive appropriate treatment, these may lead to death [10].

Due to the modern way of life, eating out has become a common practice in daily life. Eating out could be a threat to health, particularly for people with FAs, since most restaurants and cafes do not display detailed information on food ingredients on their menus. Thus, restaurants and cafes are challenging environments for people with a FA; if they are not aware of the allergens, then the risk of accidental allergen ingestion increases significantly [11]. It has been found that 21–31% of accidental allergen ingestions happen while eating in restaurants, and 13–23% of accidental allergen ingestions take place in other eating out settings, such as school and university canteens or the workplace [12].

Although studies [13,14] have been conducted on managers, food workers, and servers’ food allergy knowledge, attitudes, and practices, due to the lack of studies on the allergen awareness of consumers/the public in relation to the food items present on the menus of restaurants and cafes, the study aimed to measure the awareness of food allergies and allergens in the menus of restaurants and cafes among Saudi female university students. In addition, this study also identifies the differences in the food allergy awareness of King Saud University female students in relation to food allergens on menus in restaurants and cafes based on different variables.

## 2. Materials and Methods

### 2.1. Description of Participants

This cross-sectional study conveniently sampled Saudi students aged 18 years and above from King Saud University, taking students from all its respective colleges (Science, Health, and Humanities) and from all academic stages (bachelor’s and postgraduate studies). An email was sent to 620 students, and 398 students responded; thus, the response rate was 64.19%. A total of 19 participants were removed from the study for not fulfilling the inclusion criteria of nationality. A total of 149 participants were from the Science College, 76 from the Health college, and 154 were from the Humanities College. A total of 142 participants were from different years of post-graduation study, and 237 were from different years of bachelor study. Only 75 participants reported having a food allergy. The sample size (379) was determined using the Stephen Thompson Equation [15], as follows:

$$n=\frac{N\times p\left(1-p\right)}{\left[\left[N-1\times \left(d^{2}÷z^{2}\right)\right]+p\left(1-p\right)\right]}$$

where *N*: population size; *z*: confidence level at 95% (1.96); *d*: error proportion (0.05); and *p*: probability (50%).

### 2.2. Inclusion and Exclusion Criteria

The study included only those students whose nationality was Saudi and whose age was above 18 years of age. Three hundred ninety-eight students responded for the study; however, nineteen participants were excluded as their nationality was not Saudi.

### 2.3. Ethical Approval

The Ethical Committee for Human and Social Research at King Saud University approved this study (KSU-HE-20-700). A consent letter to participate in the study was obtained from each participant.

### 2.4. Data Collection

A pre-prepared questionnaire obtained from previous studies [16,17] was slightly modified and translated into Arabic and then reviewed by a specialized committee of three faculty members in the field of nutrition. The questionnaire was distributed to the students through university e-mails. The questionnaire consisted of two sections; the first section addresses the demographic information of the participants, including age, marital status, college, university stage, monthly income of the family, the rate of visits to restaurants or cafes per week, and food allergies. The second section contained 16 questions (close-ended) to measure the awareness and knowledge of the participants about FAs and allergens on the menus of restaurants and cafes. For example, the questions assessed the definition of food allergies, food allergens, and food allergy symptoms; the definition and prevention of cross-contact of food allergies; dealing with customers with FAs; emergency treatment procedures for people with FAs; strategies for the prevention of food allergy reactions; customer expectations towards restaurants; and preventive measures taken by customers for FAs. Correct answers were encoded as 1, and incorrect answers as 0; thus, the degree of awareness ranged from 0 to 16.

The level of awareness was divided into three categories [18], as follows:

0 to 5.33—low

>5.33 to 10.66—moderate

>10.66 to 16—high

### 2.5. Validation Process

Two types of validities, i.e., content and face validity, were determined for the questionnaire. For content validity, the first draft of the questionnaire consisting of 20 items was reviewed by a specialized committee of three faculty members in the field of nutrition. The committee members reviewed each item of the questionnaire individually and rated it based on clarity, simplicity, relevance, and obscurity. In response to the expert’s comments and opinions, four items were removed, and some questions were restructured. After the content validity, a pilot electronic survey consisting of 16 items was sent to a randomized sample of 20 female students. The aim was to test the questions’ clarity and how understandable they were, as well as to test the questionnaire’s consistency, validity, and reliability. All participants reported that the elements were clear and understandable. In this study, the value of Cronbach’s alpha coefficient using the Codder–Richardson method was 0.864, and at this level the internal consistency or reliability is considered as good, which shows that the response values for each participant across a set of questions are consistent. The validity coefficient using the Pearson Correlation coefficient was 0.92.

### 2.6. Statistical Data Analysis

Data were entered and analyzed statistically using the Statistical Package for Social Sciences (SPSS) version 22. Since the scores for the questionnaire were (1 or 0), the Kuder–Richardson 20 equation, a particular type of Cronbach’s alpha coefficient suitable for use with the scores of test questions whose scores are (1 or 0) was used to calculate the reliability of the questionnaire. A Cronbach’s alpha value of 0.7 is considered as minimally acceptable and 0.8 is considered as reliable. Moreover, the Pearson Correlation coefficient was used for calculating the validity of the questionnaire statements. In addition, to assess the awareness level, this study also identifies the differences between the levels of awareness based on different variables (college type; university stage; food allergy; the rate of visits to restaurants and cafes; age; listing all allergens on the menu in restaurants or cafes). The chi-square test was used to study the differences between the frequencies of the response to each question, and a t test was used for independent samples to study the differences that belong to two variables such as the university stage (bachelor’s and postgraduate) and FA (students with or without a FA). One-way analysis of variance (one-way ANOVA) was used to study the differences in awareness related to age, college, rate of restaurant visits, listing allergens on the menus of restaurants, cafes frequented, and three-level variables and above; then, the Scheffe test was used to find the direction of the statistically significant differences. Statistical significance was set at *p* ≤ 0.05.

## 3. Results

### 3.1. Demographic Data of the Participants

Table 1 shows the demographic data of the research sample. Most (49.08%) of the participants were 18–22 years old, and only 3.43% were more than 38 years old. In addition, 84.17% were single, while 15.83% were married. Almost 38% of the participants had a family income between 5000 and 15,000 SR. Additionally, the table shows that approximately two-thirds (62.53%) of the participants were in the bachelor’s degree program, while 37.47% of the participants were postgraduate students. Most (40.63%) were students at humanities colleges, and most of them (80.21%) did not have any FAs, while those with allergies represented 19.79%.

### 3.2. Frequency of Participants’ Visits to Restaurants and Cafes and Their Food Allergy and Food Allergen Awareness

**Figure 1:**
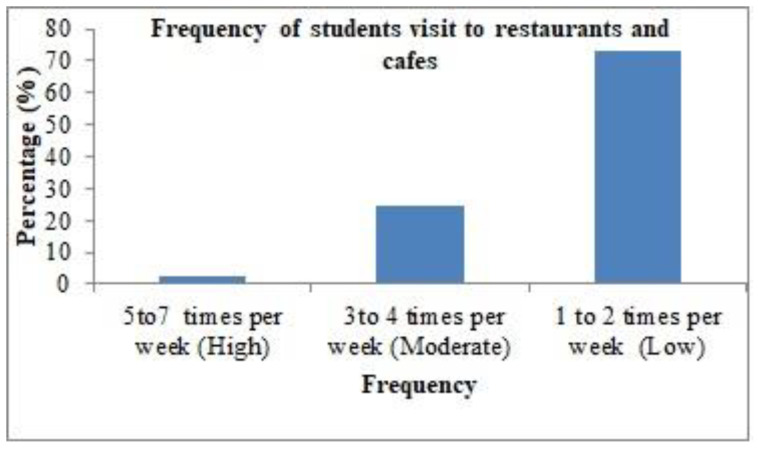
Frequency of female university students’ visits to restaurants and cafes.

**Figure 2:**
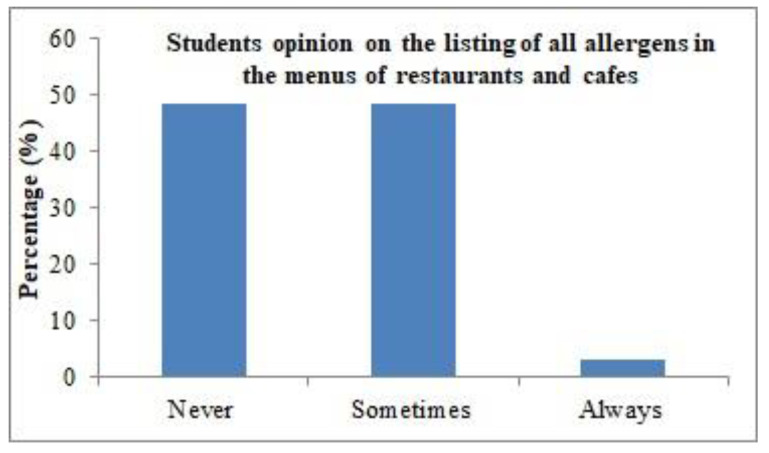
Students’ opinions on the listing of all allergens on restaurant and cafe menus.

As seen in Figure 1, the frequency of female university students’ visits to restaurants and cafes was quite low. The obtained data show that 72.82% of participants visited restaurants and cafes only once or twice per week, 24.80% visited three to four times per week, while only 2.37% visited restaurants and cafes five to seven times a week. Figure 2 depicts the opinion of students on the listing of all allergens on the menu in restaurants and cafes. It was observed that 48.55% of participants considered that food allergens are never listed on the menus at restaurants and cafes, while 48.28% considered that food allergens are sometimes listed on menus. On the other hand, 3.17% of the sample reported that all food allergens are always listed on menus.

Table 2 depicts the food allergy and food allergen awareness of participants in relation to restaurant and cafe menus. It was noted that 87.6% of participants were aware that food allergy reactions occur immediately or within a few hours after the food is consumed, and almost 74% responded correctly that the respiratory system, gastrointestinal tract, and skin are all affected by food allergy reactions. Most of the participants were aware of the top food allergens and best possible treatment for controlling a severe allergic reaction. Almost 61% correctly responded that potato is not among the top food allergens, 51.19% correctly identified the risky food items for a person who has a FA, and 67.5% were aware that epinephrine is the best possible treatment for controlling a severe allergic reaction. Almost 60% of participants were aware of the correct definition of allergen cross-contact, and 81.5% believed that avoiding the food that causes the allergic reaction is the best possible way to prevent a reaction. Seventy-seven percent of participants felt that all restaurants and cafes should provide allergen-free menu options, mark the eight major food allergens on the menus, and list all the ingredients of food and drinks. The study also showed that 85.75% of participants believe that each restaurant and cafe should have procedures in place that allow food allergy sufferers to more easily choose from the menu in order to avoid allergens that may cause an allergic reaction.

Table 3 shows a statistically significant difference in favor of correct answers at the 0.01 level between the frequency of correct answers and the frequency of incorrect answers in question numbers 1, 2, 4, 6, 7, 8, 9, 12, 14, 15, and 16. This signifies that the number of correct responses to the above-mentioned questions was statistically more significant than the number of incorrect answers. This outcome indicates that the research samples from the female campus of King Saud University possess high awareness of food allergens in the restaurant and cafe menus included in these 11 questions. For both questions 3 (The body reacts negatively to which of the following foods to cause FAs?) and 11 (Why can fried foods be dangerous for people with FA?), a statistically significant difference at the 0.01 level between the frequency of correct answers and the frequency of incorrect answers in favor of the number of incorrect answers was observed. This means that the number of incorrect answers to these two questions was higher and statistically more significant than the number of correct answers. This suggests that the research samples had low awareness of food allergens on the menus of restaurants and cafes. There were statistically insignificant differences at the 0.01 level between the frequency of correct answers and the frequency of incorrect answers to questions 5, 10, and 13, indicating that the research sample was moderately aware of food allergens on the menus of restaurants and cafes. At the 0.01 level, there was a statistically significant difference in the frequencies of students’ awareness about food allergens on restaurant and cafe menus (high, moderate, low) in favor of students with a high level of awareness. This suggests that the number of students with a high level of awareness was significantly higher than the number of students with moderate and low levels of awareness. The results depicts that the overall average awareness was 10.90, which falls within the high range (>10.66 to 16—high).

Table 4 shows the differences in food allergy awareness due to the age variable. A statistically significant difference (*p* ≤ 0.01) in food allergy awareness in relation to restaurant and cafe menus was noticed due to this variable. According to the Scheffe test results, KSU female students aged between 23 and 27 years old were more aware of food allergens on restaurant and cafe menus than female students aged between 18 and 22 years old, and this difference was statistically significant at the 0.05 level.

### 3.3. N-Number

Table 5 shows the differences in food allergy awareness due to the college variable. Statistically significant differences (*p* ≤ 0.01) were noticed in relation to awareness about food allergens on restaurant and cafe menus due to this variable. Based on the Scheffe test findings, it is clear that the science college and health college students were more aware of food allergens on restaurant and cafe menus than students at humanities colleges, and this difference was statistically significant at the 0.05 level. However, a statistically insignificant difference (*p* > 0.01) was observed in the awareness of students regarding food allergens on restaurant and cafe menus among students from the health and science colleges.

Table 6 shows a statistically significant difference (*p* ≤ 0.05) between the mean scores of the bachelor’s and postgraduate female students in relation to awareness about food allergens on restaurant and cafe menus, with the mean score of the postgraduate students being higher, i.e., female postgraduate students’ awareness about food allergens on restaurant and cafe menus was statistically significantly higher than that of female bachelor’s students.

Table 7 shows statistically insignificant (*p* > 0.05) differences in the awareness among students about food allergens on restaurant and cafe menus due to the restaurant/cafe visit frequency (high, medium, and low) variable.

Table 8 shows a statistically insignificant difference (*p* > 0.05) between the mean scores of students with and without a FA in the level of awareness about food allergens on restaurant and cafe menus, indicating the similarity in the level of awareness about food allergens on restaurant and cafe menus in female students both with and without a FA.

Table 9 shows a statistically significant difference in awareness of food allergens on restaurant and cafe menus (*p* ≤ 0.01) due to the variable of listing all allergens on the menu. Based on the Scheffe test results, it is evident that visiting restaurants and cafes that sometimes list all allergens on the menu increases the statistically significant (at the 0.05 level) outcome of female students’ awareness of food allergens compared to visiting restaurants and cafes that never list all allergens on the menus.

## 4. Discussion

FA prevalence is an increasingly important public health issue. Nowadays, especially among youth, dining outside the home at restaurants and cafes is a popular pastime, and dependency on and craving of convenience foods and fast foods have rapidly increased [19]. Even though the event of dining out provides an opportunity for social bonding, relaxation, and convenience, it may cause health problems for individuals with food allergies [20]. A survey conducted in 2020 across eight European countries on the effects of having a peanut allergy reported that around 89% of subjects felt restricted by the allergy; in terms of dining out and selecting a destination, 65% felt isolated while 90% felt tense about the whole procedure [21]. For a variety of reasons, such as the improper handling of food allergens, inadequate employee awareness of food allergies, hidden allergens, and miscommunication between employees and customers, food allergy reactions have commonly occurred in restaurants or commercial food service establishments [17]. Therefore, an awareness of food allergies is crucial to prevent accidents. Bearing this in mind, the present study assessed people’s awareness of food allergies and allergens on the menus of restaurants and cafes among female university students.

Similar to this study, a previous study assessing food service employees’ food allergy knowledge, attitudes, practices, training, and training needs reported that the majority of the respondents were 18–25 years old [16]. In this study, almost 20% of the participants were allergic to at least one food, which is in accordance with a previous study [22], which reported that almost 15% of participants were allergic to at least one food, but that their allergy did not affect their life significantly. Another study indicated that responders were well aware of cow’s milk allergies [23]. The current findings are also congruent with the findings of Choi et al. in 2012 [16], whose study targeted students and employees at on-campus residential restaurants and cafes and found that respondents were aware and highly knowledgeable about food allergy definitions and how to deal with clients who have food allergies; however, most of the responders were not able to identify the common food allergens from the list provided to them and were not aware of the best treatment available for controlling a severe food allergy reaction. Barnett et al. (2011) reported that food prepared exclusively with safe ingredients can still cause reactions if prepared using utensils that were in contact with allergens [11]. Awareness of allergens is of great significance, because a lack of awareness may cause cross-contact with an ingredient without the realization that the ingredient is a major allergen, which can in turn cause serious harm to people with allergies [24].

In this study, the overall awareness was 10.90, which falls within the high range. This indicates that the research sample possesses a high level of awareness regarding the food allergens on restaurant and cafe menus. The reason for the high level of awareness among the research sample could be that the participants were educated. Various studies in different countries have reported different levels of food allergy knowledge. Studies in Iran [25] and Germany [13] have reported suboptimal levels of food allergy awareness among the study samples, while in contrast, some studies in Turkey [14,26] reported a moderate level of food allergy awareness. In a study examining differences in parent knowledge about pediatric food allergies [27], suboptimal FA knowledge among parents was reported. This may be due to the fact that parents can forget the information supplied by doctors, which leads to a lack of information application. Therefore, doctors and parents of patients should use methods that help them to understand and maintain instructions, such as an educational pamphlets and recording the information [28,29]. Social media is another source of information that can influence awareness. The findings of a previous study indicated that apart from other people’s food allergy experiences and advice, social media allows people to access up-to-date information regarding food allergies [30].

A previous study [22] showed that young people aged 15 to 20 had a high level of awareness of the basics of food allergies, while another study on adolescents reported that adolescents aged 13 to 20 had only basic knowledge of allergies [31]. The results of our study showed that awareness about food allergies and allergens on restaurant and cafe menus among KSA female students aged 33 to 37 was higher than that of female students aged 18 to 22. This outcome suggests that the older the students are, the higher their level of awareness about food allergens on restaurant and cafe menus. A study of pediatricians found that their knowledge levels were significantly related to their age, with older pediatricians having higher knowledge of food allergies [32]. In contrast, another study revealed an insignificant difference in food allergy knowledge based on age [33].

The difference in understanding between health/science colleges and humanities colleges might be attributed to the universities’ teaching curriculum. This could also be due to the training provided to students at health and science colleges, where theoretical information is applied practically, thereby increasing their experience and knowledge. In a previous study on fifth-semester medical students, it was found that these students possess a good level of knowledge of food allergic reactions (up to 85.2%); indeed, medical students represent a good way to convey information about food allergic reactions because they are future doctors [34]. In the same vein, another study found that science students had significantly higher levels of nutritional knowledge than humanities and social sciences students, because science students are more likely to be exposed to nutritional information in their studies than humanities and social sciences students [35]. In contrast, a study on physical education students [36] found that the participants had insufficient knowledge of nutrition, probably due to a lack of application of the information gained through study and the low usage of this information in behavioral practices and daily habits.

Female postgraduate students’ level of awareness about food allergies and allergens on restaurant and cafe menus was significantly higher than that of female bachelor’s students. This might be because the higher the educational degree, the greater the individual’s level of awareness, which is consistent with the findings of a study that found that participants with a fellowship and a bachelor’s degree had a higher awareness of food allergies than their counterparts [14]. Another study found that education level had a significant impact on food allergy knowledge; these findings might indicate that higher education levels are related with better skills, providing respondents with more efficient methods to fully understand food allergy information [33]. In addition, a previous study was conducted to assess trans-fat knowledge among a group of health-interested adults to make recommendations regarding dietary education needs; the findings showed that level of education was associated with the degree of trans-fat knowledge.

Those participants with bachelor’s, master’s, and PhD degrees outperformed those with education levels lower than a bachelor’s degree [37]. This outcome is in line with the findings of a study conducted in Iraq, which mentioned that the level of awareness was related to the level of education [38]. Our results are also consistent with another study that examined dietitians’ knowledge of the biological roles of inorganic food nitrates in the United Kingdom and explored potential differences based on participants’ educational levels. Individuals with different levels of education had significantly varying levels of knowledge about inorganic nitrates, with those having master’s or PhD degrees having greater levels of knowledge than those with bachelor’s degrees [39].

In this study, statistically insignificant differences in the awareness about food allergens on restaurant and cafe menus among students due to the variable of restaurant/cafe visit frequency (high, medium, and low) were observed. One reason for this might be that the research sample consisted of participants from the same university, which increased the possibility that they often visit the same restaurants in the same city. Another reason might be that the food providers and employees have sufficient awareness, which is reflected in the awareness of female students who visit those restaurants and cafes. To the best of our knowledge, no study has addressed the effect of the frequency of weekly visits to restaurants and cafes on food allergen awareness; thus, we consider it necessary to conduct further research on this variable. In this study, no difference in the awareness among participants with or with or without food allergies about food allergens on restaurant and cafe menus was noted. It is possible that this outcome was due to enhanced levels of attention and consciousness among people with food allergies as a result of their anxiety about food allergy complications. People without food allergies may have a high level of awareness due to their relatives or friends who have food allergies. In contrast, a previous study found a difference in awareness levels among those with and without food allergies, showing that people with food allergies were more aware than those without food allergies [22].

The high levels of awareness about food allergens is due to the Saudi Food and Drug Authority (SFDA) enforcement of legislation requiring restaurants and cafes to provide information on allergens on menus. Furthermore, our findings are in agreement with those of another study [40], which found that 58.9% of the participants reported observing allergen information on menus after the SFDA enforced the legislation. Despite the EU-wide legislation introduced in December 2014 requiring restaurant servers to provide written and verbal information about one or more of the fourteen most common food allergens in their food, some studies have found that food allergens are not listed on menus or on other documents, such as on restaurant signs [41]. These findings are concerning, because food-allergic customers often rely on written information about food allergens while dining out to help them avoid potential allergen exposure [42,43]. Therefore, enhanced efforts by restaurant and food service employees are needed to build confidence in food-allergic customers and to provide accurate and reliable information about the risks of food allergens [44].

## 5. Conclusions

The current study investigated the awareness of food allergies and allergens in the menus of restaurants and cafes among Saudi female university students. Overall, the participants were knowledgeable about food allergies and allergens on restaurant and cafe menus. It was observed that various factors such as age, education, and type of college attended have been associated with higher awareness in students. The outcomes of this study further show that most of the participants were cognizant of the preventive measures needed to ensure their own safety. Although the SFDA has enforced legislation specifying that restaurants and cafes should provide information on allergens on menus, most of the students have reported that restaurants’ or cafes’ menus either do not mention or provide only a small amount of information about allergens; we therefore wish to motivate restaurants and cafes to provide more information about allergens on their menus.

Students can play an active role in improving consumer safety and health. University students can encourage and educate other students, and they can also increase the awareness of community workers and the public by providing training on food allergies and their management.

The major limitation of this study was that this study comprised only university students that were from one university, which might be responsible for the overrepresentation of awareness; thus, the results from the survey cannot be generalized beyond our participants. Only 19.79% of participants in this study had an FA, so the results may not cover the entire spectrum of opinions in relation to people with food allergies.

## Figures and Tables

**Table 1:** Demographic data of the participants (*n* = 379).

Variable	Number	Percentage
Age (years)		
18 to 22	186	49.08%
23 to 27	103	27.17%
28 to 32	60	15.83%
33 to 37	17	4.49%
38 and above	13	3.43%
Marital Status		
Married	60	15.83%
Single	319	84.17%
Monthly Income (SR)		
<5000	65	17.15%
5000 to <15,000	143	37.73%
15,000 to <25,000	87	22.96%
>25,000	84	22.16%
Education		
Bachelor’s	237	62.53%
Post-graduate	142	37.47%
College		
Science	149	39.31%
Health	76	20.06%
Humanities	154	40.63%
Food Allergy		
Present	75	19.79%
Absent	304	80.21%

**Table 2:** Food allergy and food allergen awareness of participants in relation to restaurant and cafe menus (*n* = 379).

Awareness Questions	N (%)
1. What is the average time it takes for a food allergy reaction to occur after the food has been consumed?	
(a) Immediately or within a few hours after the food is consumed *	332 (87.6)
(b) 24 h after the food is consumed	40 (10.55)
(c) 36 h after the food is consumed	3 (0.79)
(d) 48 h after the food is consumed	4 (1.06)
2. When a food allergy reaction occurs, which body system is affected?	
(a) Respiratory system	10 (2.64)
(b) Gastrointestinal tract	43 (11.35)
(c) Skin	46 (12.14)
(d) All of the above *	280 (73.87)
3. The body reacts negatively to which of the following foods, thus causing food allergies?	
(a) Carbohydrates	99 (26.12)
(b) Proteins *	159 (41.96)
(c) Fats	50 (13.19)
(d) Trans-fatty acids	71 (18.73)
4. Which one of the following does not belong among the top food allergens?	
(a) Tofu	114 (30.08)
(b) Shrimp	19 (5.01)
(c) Wheat	15 (3.96)
(d) Potato *	231 (60.95)
5. Which of the following items are risky for those who have food allergies?	
(a) Fried foods	23 (6.01)
(b) Desserts	19 (5.01)
(c) Complex dishes with many ingredients	143 (37.73)
(d) All of the above *	194 (51.19)
6. Mark all symptoms of food allergy reactions exhibited in adults with a milk allergy who have accidentally consumed a food containing milk ^#^	
(a) Shortness of breath *	234 (61.74)
(b) Headache *	100 (26.39)
(c) loss of consciousness *	72 (19.00)
(d) Nausea *	297 (78.36)
(e) Vomiting *	265 (69.92)
(f) Sneezing/Cough *	93 (24.53)
(g) Rash *	264 (69.66)
(h) Hyperactivity	6 (1.58)
(i) Epigastric pain *	305 (80.47)
7. Is it true that a person can die from an allergic reaction to food?	
(a) True *	328 (86.54)
(b) False	51 (13.46)
8. From the given options, select the best possible treatment for controlling a severe food allergic reaction	
(a) Benadryl	45 (11.87)
(b) Sudafed	23 (6.07)
(c) Epinephrine *	256 (67.55)
(d) Pseudoephedrine	55 (14.51)
9. Which of the following is the correct definition of allergen cross-contact?	
(a) Contact between raw and cooked foods	27 (7.12)
(b) Contact between allergen-containing foods and non-allergen-containing foods*	226 (59.63)
(c) Contact between allergen-containing foods and raw meat	17 (4.49)
(d) Contact between allergen-containing foods and dairy products	109 (28.76)
10. Which of the following practices could cause cross-contact?	
(a) Using the same utensil for preparing allergen-containing food and allergen-free food	152 (40.1)
(b) Not washing hands but using a fresh pair of gloves before handling the allergen-free food	4 (1.06)
(c) Preparing allergen-free food on a countertop that has not been thoroughly cleaned and sanitized	17 (4.49)
(d) All of the above *	206 (54.35)
11. Why can fried foods be dangerous for people with food allergies?	
(a) The high-fat content in fried foods makes allergic reactions worse	116 (30.61)
(b) Frying changes the chemical structure of foods	67 (17.68)
(c) Cross-contact with other food proteins can occur if the oil is used for cooking other foods *	157 (41.42)
(d) The high starch content makes allergic reactions worse	39 (10.29)
12. The basic principle of food allergy treatment is to avoid the food that causes allergic reaction	
(a) True *	309 (81.53)
(b) False	70 (18.47)
13. Modern medicine can cure food allergies	
(a) True	198 (52.24)
(b) False *	181 (47.76)
14. Restaurants and cafes should	
(a) Provide allergen-free menu options	17 (4.49)
(b) Mark the eight major food allergens on the menus	55 (14.51)
(c) List all ingredients of food and drinks	15 (3.96)
(d) All the above *	292 (77.04)
15. Customers with severe food allergies should	
(a) Always bring “back-up” food if the restaurants cannot provide allergen-free food	13 (3.43)
(b) Always carry an epinephrine auto-injector when dining out	37 (9.76)
(c) Tell restaurant employees about their food allergies	48 (12.67)
(d) All the above *	281 (74.14)
16. The menu should include options for those with food allergies to avoid allergens that might cause an allergic reaction	
(a) True *	325 (85.75)
(b) False	54 (14.25)

* Indicates correct answers. ^#^ Percentage may not be 100% due to multiple responses.

**Table 3:** Study on the frequencies of differences between the response to each question and the participants’ awareness of food allergies and allergens on the menus of restaurants and cafes.

Question No.	Correct Response	Incorrect Response	Chi Square Test	Sig. Level	Mean	Level of Awareness			Chi Square Value	Sig. Level
						High	Moderate	Low		
1	332	47	214.3	0.01	0.88	226	147	6	196.6	0.01
2	280	99	86.4	0.01	0.74					
3	159	220	9.82	0.01	0.42					
4	231	148	18.2	0.01	0.61					
5	194	185	0.2	0.64	0.51					
6	373	6	355.4	0.01	0.98					
7	328	51	202.5	0.01	0.87					
8	256	123	46.7	0.01	0.68					
9	226	153	14.1	0.01	0.60					
10	206	173	2.9	0.09	0.54					
11	157	222	11.1	0.01	0.41					
12	309	70	150.7	0.01	0.82					
13	181	198	0.8	0.38	0.48					
14	292	87	110.9	0.01	0.77					
15	281	98	88.4	0.01	0.74					
16	325	54	193.8	0.01	0.86					
	Average of total awareness				10.90	Total Awareness Level = High				

Model: Chi square; *p* ≤ 0.01.

**Table 4:** Study on the differences in the awareness of food allergies and allergens on the menus of restaurants and cafes according to age.

Age	N	Mean	Std. Dev.	18–22	23–27	28–32	33–37	F Value	Sig. Level
18–22	186	10.47	2.29	-				4.07	0.01
23–27	103	11.47	2.54	1.00 *	-				
28–32	60	10.77	2.36	0.30	0.70	-			
33–37	17	11.94	3.40	1.47 *	0.48	1.17	-		
38 and over	13	11.77	2.68	1.30	0.30	1.00	0.17		

Model: One-way analysis of variance (ANOVA); Test: Scheffe test. * Indicates that the difference between the two means is statistically significant at the (0.05) level. Std. dev.—Standard deviation.

**Table 5:** Study on the differences in the awareness of food allergies and allergens on the menus of restaurants and cafes according to college type.

College	Number	Mean	Std. Dev.	Science College	Health College	F Value	Sig. Level
Science college	149	11.21	2.38	-		11.99	0.01
Health college	76	11.70	2.02	0.49	-		
Humanities College	154	10.19	2.61	1.02 *	1.51 *		

Model: One-way analysis of variance (ANOVA); Test: Scheffe test. * Indicates that the difference between the two means is statistically significant at the (0.05) level. Std. dev.—Standard deviation

**Table 6:** Study on the awareness of food allergies and allergens on the menus of restaurants and cafes according to qualification level (undergraduate and graduate).

Dependent Variable	Independent Variable	Number	Mean	Std. Dev.	T Value	Sig. Level
Awareness level	Bachelor’s students	237	10.68	2.32	2.17	0.05
	Postgraduate students	142	11.25	2.70		

Model: The *t*-test results for the two independent samples; *p* ≤ 0.05. Std. dev.—Standard deviation

**Table 7:** Study on the differences in awareness of food allergies and allergens on the menus of restaurants and cafes according to the frequency of visits to restaurants and cafes.

Frequency of Visits to Restaurants and Cafes	Number	Mean	Std. Dev.	F Value	Sig. Level
High (5–7 times/week)	9	10.56	2.92	0.12	0.89
Moderate (3–4 times/week)	94	10.85	2.31		
Low (1–2 times/week)	276	10.92	2.53		

Model: One-way analysis of variance (ANOVA); Std. dev.—Standard deviation; *p* ≤ 0.05.

**Table 8:** Study on the differences in the awareness of food allergies and allergens on the menus of restaurants and cafes for female students with and without food allergies.

Dependent Variable	Independent Variable	Number	Mean	Std. Dev.	T Value	Sig. Level
Awareness level	Students with food allergies	75	10.72	2.17	0.69	0.49
	Students without food allergies	304	10.94	2.56		

Model: *t*-test for the two independent samples; *p* ≤ 0.05. Std. dev.—Standard deviation

**Table 9:** Study on the differences in the awareness of food allergies and allergens in the menus of restaurants and cafes according to the variable of listing all food allergens on the menu.

Listing of Food Allergens	Number	Mean	Std. Dev.	Never	Sometimes	F Value	Sig. Level
Never (10% or less)	184	10.38	2.63			8.95	0.01
Sometimes (40% to 60%)	183	11.44	2.25	1.07 *			
Always (80% to 100%)	12	10.58	1.83	0.21	0.86		

Model: One-way analysis of variance (ANOVA); Test: Scheffe test. * Indicates that the difference between the two means is statistically significant at the (0.05) level. Std. dev.—Standard deviation

## Data Availability

The data presented in this study are available on request from the corresponding author.
